# AID downregulation is a novel function of the DNMT inhibitor 5-aza-deoxycytidine

**DOI:** 10.18632/oncotarget.1319

**Published:** 2013-11-25

**Authors:** Chiou-Tsun Tsai, Pei-Ming Yang, Ting-Rong Chern, Shu-Hui Chuang, Jung-Hsin Lin, Lars Klemm, Markus Müschen, Ching-Chow Chen

**Affiliations:** ^1^ Department of Pharmacology, College of Medicine, National Taiwan University, Taipei, Taiwan; ^2^ School of Pharmacy, College of Medicine, National Taiwan University, Taipei, Taiwan; ^3^ Research Center for Applied Sciences, Academia Sinica, Taipei, Taiwan; ^4^ Institute of Biomedical Science, Academia Sinica, Taipei, Taiwan; ^5^ Department of Laboratory Medicine, University of California San Francisco, San Francisco, California

**Keywords:** AID, 5-aza-CdR, Zebularine, DNMT1

## Abstract

Activation-induced cytidine deaminase (AID) was originally identified as an inducer of somatic hypermutation (SHM) and class switch recombination (CSR) in immunoglobulin genes. However, AID can also cause mutations in host genes and contribute to cancer progression and drug resistance. In this study, molecular docking showed the interaction of free 5-aza-CdR and Zebularine (Zeb) with AID. However, only 5-aza-CdR-incorporated ssDNA bound to the active site of AID and inhibited AID expression through proteasomal degradation. 5-aza-CdR demonstrated cytotoxicity against AID-positive and -negative hematopoietic cancer cells. In contrast, Zeb exhibited a cytotoxic effect only in AID-negative cells due to its inability to inhibit AID expression. This differential effect might be due to the DNMT1 stabilization induced by AID, thus restricting the ability of Zeb to deplete DNMT1 and induce tumor suppressor genes (TSGs), such as p21, in AID-positive cells. Moreover, the in vivo anticancer effect of 5-aza-CdR but not Zeb in AID-positive hematopoietic cancer cells was demonstrated. The study not only displays the association of AID and DNMT1 and identifies a novel biological function of AID, but also provides novel information regarding the use of DNMT inhibitors to treat AID-positive hematopoietic cancers.

## INTRODUCTION

AID, encoded by the *AICDA* gene, belongs to the apolipoprotein B-editing catalytic polypeptide (APOBEC) family and was originally described as a B cell–specifc factor unique to activated germinal center B cells. During CSR, AID is recruited to the switch region to deaminate the nucleoside cytidine and convert it to uridine, causing DNA point mutations and double strand breakage [1]. This activity is essential for SHM and CSR, which generates immunoglobulin diversity after V(D)J recombination [2]. In contrast to the favorable role of AID in the immune system, AID can cause chromosomal translocations and/ or mutations in proto-oncogenes, thus promoting tumor formation [3]. For example, AID induces double strand breaks in the *c-myc* gene, resulting in its translocation to the *Ig* loci and uncontrolled expression of c-Myc in Burkett's lymphoma [4, 5].

AID also plays an essential role in the progression of Philadelphia-positive (Ph+) leukemias, including chronic myeloid leukemia (CML) and Ph+ acute lymphoblastic leukemia (ALL) [6, 7]. The Ph chromosome originates from a translocation between the *c-abl* on chromosome 9 and the *bcr* gene on chromosome 22, leading to a BCR/ABL1 fusion protein. The forced expression of the Abelson tyrosine kinase ABL1 can phosphorylate a wide range of substrates that regulate cell proliferation, differentiation, migration, survival, and DNA repair and drive the pathogenesis of Ph+ leukemias [8]. Clinically, CML follows a triphasic pattern of chronic, accelerated, and blast crisis. The majority of patients (85%) in the chronic phase will progress to the accelerated phase and blast crisis if untreated [9]. AID is expressed in a subset of CML patients in lymphoid blast crisis, which promotes the genetic instability of tumor suppressors and DNA repair genes through point mutations and copy number alterations. In addition, AID mutates BCR-ABL1, providing a rationale for the rapid development of imatinib resistance in blast crisis progression [6].

AID is also expressed in Ph+ ALL patients, who show an increased mutation frequency of oncogenes and TSGs, such as *MYC*, *BCL6*, and *p16*, which may be relevant to the unfavorable prognosis in this subset of ALL [7]. Bone marrow isolated from wild-type and AID knockout mice were transduced with BCR-ABL1 to induce BCR-ABL1-driven ALL; then, ALL cells were transplanted into the mice. The mice engrafted with AID-/- ALL cells showed prolonged survival compared with those transplanted with AID+/+ ALL cells. Molecular analyses showed that AID-/- ALL cells had lower frequencies of amplification, deletion and point mutation in non-Ig genes, such as Pax5 and Rhoh, and failed to repress TSGs including Rhon, p21 and Blnk. The results indicate that AID may be an oncoprotein that accelerates the evolution of ALL through aberrant hypermutation and TSG downregulation [10]. A study has also shown that AID can induce B-lymphoma/leukemia in a bone marrow transplantation mouse model, and its activity to induce CSR and SHM is essential for lymphomagenesis [11].

The cytidine ribose nucleoside analogue 5-aza-CR (5-azacytidine) was initially identified as a potential anticancer drug and was subsequently shown to be a DNA methyltransferase (DNMT) inhibitor [12]. After incorporation into DNA, 5-aza-CR is recognized by DNMT1 to form stable covalent protein-DNA adducts. DNMT1 are trapped and degraded, leading to rapid protein diminish as early as 24 hours treatment [13, 14]. Eventually, DNA demethylation occurs, and then TSGs are induced to inhibit cancer cell proliferation [15]. 5-aza-2-deoxycytidine (5-aza-CdR), the deoxyribose analogue of 5-aza-CR, was subsequently developed [16]. Cytidine deaminase (CDA) is a key enzyme in the pyrimidine salvage pathway, catalyzing the deamination of cytidine and deoxycytidine into uridine and deoxyuridine. CDA also deaminates cytidine analogues, including 5-aza-CR and 5-aza-CdR, to reduce their stability [17, 18]. Another DNMT inhibitor, 1-(β-D-ribofuranosyl)-2(1H)-pyrimidinone (zebularine; Zeb), is a cytidine analogue that contains a 2-(1*H*)-pyrimidinone ring. Zeb was originally synthesized as a CDA inhibitor [19]. Because both CDA and AID can catalyze cytidine deamination, AID might also be targeted by cytidine analogues.

In this study, molecular docking analysis showed the interaction of both free 5-aza-CdR and free Zeb with AID. However, only 5-aza-CdR-incorporated single-strand DNA (ssDNA) bound to the active site of AID and inhibited AID expression through proteasomal degradation. The cytotoxicity of 5-aza-CdR was observed in both AID-positive and -negative hematopoietic cancer cells. In contrast, Zeb cytotoxicity was only observed in AID-negative cells due to its inability to inhibit AID expression. This differential effect might be due to the association and stabilization of DNMT1 by AID, thus restricting the ability of Zeb to deplete DNMT1 and induce TSGs, such as p21, in AID-positive cells. Moreover, the anticancer effect of 5-aza-CdR but not Zeb on AID-positive hematopoietic cancers was also demonstrated in vivo. The study not only displays the association of AID and DNMT1 and identifies a novel biological function of AID, but also provides a novel role of a DNMT inhibitor for treating AID-positive hematopoietic cancers.

## RESULTS

### Molecular docking of DNMT inhibitors to AID

**Figure 1:**
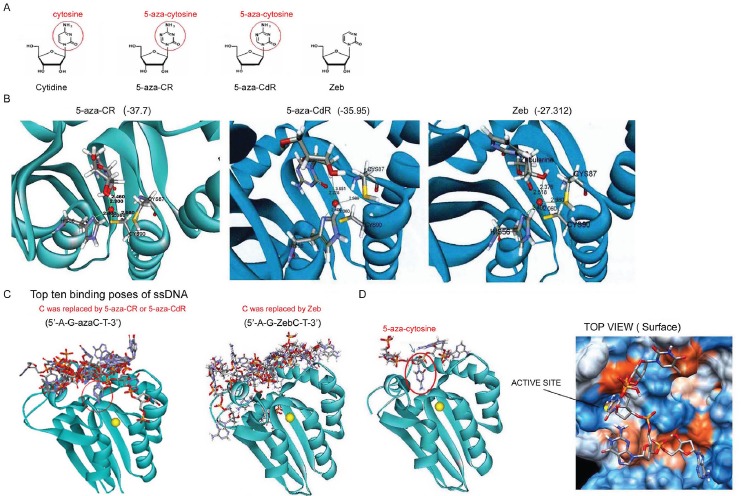
Molecular docking of DNMT inhibitors to AID (A) Molecular structures of cytidine and its analogs. (B) The docking sites of DNMT inhibitors were analyzed using CDOCKER, and the best structures were selected based on the lowest CDOCKER_ INTERACTION_ENERGY for each ligand. (C) 5-aza-CdR and Zeb were incorporated into the DNA sequence (5'-AGCT-3') built with “Build and Edit Nucleic Acid” tools in Discovery Studio 2.55, and the binding modes were further analyzed. The top ten binding structures are shown. (D) The best insertion model is presented by a ribbon (left panel) and surface model (right panel).

A previous study showed that the DNMT inhibitor Zeb, a cytidine analogue, is a competitive inhibitor of CDA due to its lack of a 4-amino group on the cytosine (Fig. 1A) [20]. Because AID also catalyzes cytidine deamination, we hypothesized that cytidine analogues, such as 5-aza-CR, 5-aza-CdR, and Zeb, might inhibit AID. Molecular modeling simulation was performed to test this hypothesis. To our knowledge, the crystal structure of AID has not yet been solved. Because the sequences of AID and the APOBEC2 fragment shared 51.0% similarity and 33% identity (suppl. Fig. S1A), the AID structure was predicted based on the crystal structure of APOBEC2 [21] (suppl. Fig. S1B). The proposed binding modes of 5-aza-CR, 5-aza-CdR, and Zeb are illustrated in Figure 1B. Free 5-aza-CR, 5-aza-CdR, and Zeb bound to the active site of AID and interacted with the zinc ion that is crucial for the enzymatic reaction by the 2',3' hydroxyl group and the 2' carbonyl group (Fig. 1B and suppl. S1C). Based on CDOCKER interaction energy, the binding capacity of 5-aza-CR (-37.7) and 5-aza-CdR (-35.95) was higher than that of Zeb (-27.312). These cytidine analogues are incorporated into nucleic acids after entering the cell membrane [13]. A previous study has shown that AID targets the immunoglobulin H (IgH) switch region, which contains 5'-AGCT-3' repeats in its core [22]. Therefore, 5'-AGCT-3' ssDNA, in which the cytidine base was substituted by the respective azacytidine (5'-AG-azaC-T-3') or Zeb (5'-AG-ZebC-T-3'), was analyzed by molecular docking. The 10 predicted binding modes are illustrated in Figure 1C. Compared with 5'-AG-ZebC-T-3' (Fig. 1C, right panel), 5'-AG-azaC-T-3' was closer to the catalytic site of AID, and both of them inserted into the active site (Fig. 1C, left panel). The best insertion model showed that the substituted 5-aza-cytosine inserted exactly into the small active site (Fig. 1D), while the remainder of the ssDNA interacted with the polar surface (as indicated by the orange color) of AID (Fig. 1D, right panel). These findings suggest that only azacytidine-incorporated ssDNA could interact with AID.

### 5-aza-CdR but not Zeb destabilizes AID through ubiquitin-proteasomal degradation

**Figure 2:**
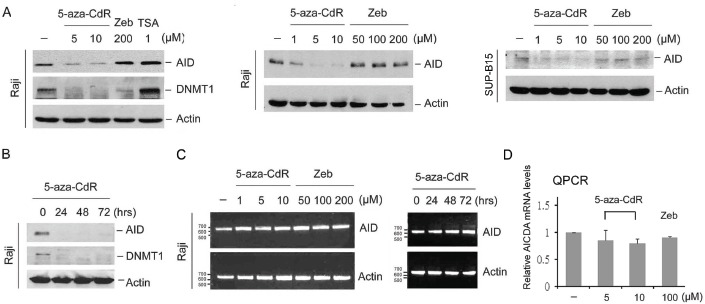
5-aza-CdR downregulated AID Raji cells and SUP-B15 were treated with 5-aza-CdR (1-10 μM), Zeb (50-200 μM), or TSA (1 μM) for 4 days (A) or 5-aza-CdR (5 μM) for 24, 48, and 72 hrs (B). The protein expression levels of AID, DNMT1 and actin were analyzed through immunoblotting. (C) Raji cells were treated with 5-aza-CdR (1-10 μM) or Zeb (50-200 μM) for 4 days (left panel) or 5-aza-CdR (5 μM) for 24, 48, and 72 hrs (right panel). The mRNA levels of AICDA and actin were analyzed through RT-PCR. (D) Raji cells were treated with 5-aza-CdR (5-10 μM) or Zeb (100 μM) for 4 day. The relative mRNA levels of AICDA were analyzed through QRT-PCR

Once incorporated into DNA, azacytidine traps DNMT and triggers its degradation [14]. Because azacytidine- but not Zeb-substituted ssDNA interacted with AID (Fig. 1C), we proposed that 5-aza-CdR and 5-aza-CR would also trigger AID degradation. Therefore, the inhibitory effect of DNMT inhibitors on AID expression was examined in Burkitt's lymphoma Raji and Ph+ ALL SUP-B15 cells, which possess AID. 5-aza-CdR, 5-aza-CR, and Zeb all downregulated DNMT1 expression [13]; however, only 5-aza-CdR and 5-aza-CR inhibited AID expression (Fig. 2A, 2B and suppl. Fig. S2A). However, *AICDA* mRNA was not significantly affected by 5-aza-CdR (Fig. 2C and 2D), indicating that 5-aza-CdR might inhibit AID expression through post-transcriptional regulation.

**Figure 3:**
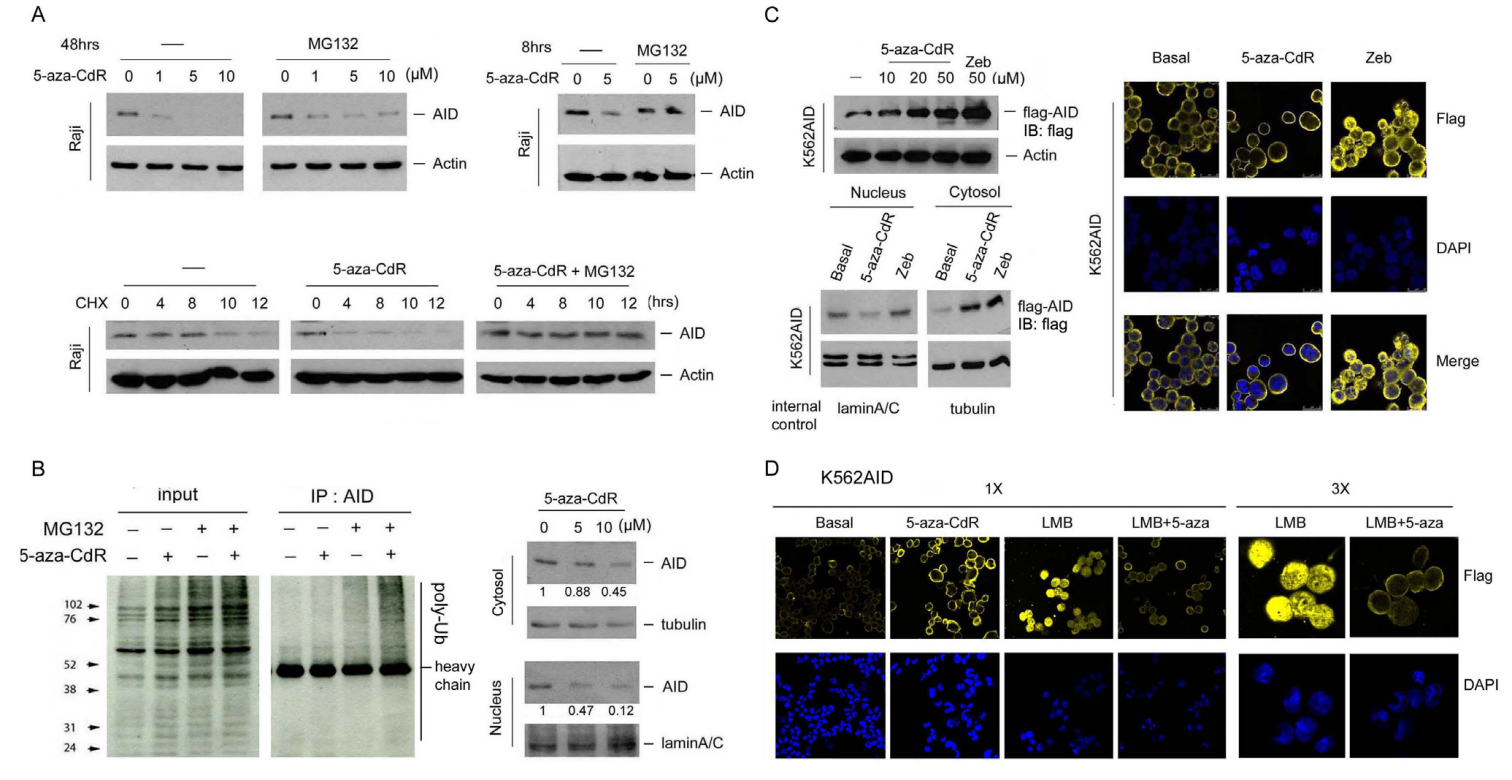
5-aza-CdR reduced the protein stability of nuclear AID (A) Upper-left panel: Raji cells were treated with 5-aza-CdR (1-10 μM) for 40 hrs, and MG132 (10 μM) was added for another 8 hrs. Upper-right panel: Raji cells were co-treated with 5-aza-CdR (5 μM) and MG132 (10 μM) for 8 hrs. The protein expression levels of AID and actin were analyzed through immunoblotting. Lower panel: Raji cells were pretreated with 5-aza-CdR (5 μM) for 16 hrs. Then, the cells were exposed to cycloheximide (20 μM) or cycloheximide/ MG132 (10 μM) for 4, 8, 10, and 12 hrs. The protein expression levels of AID and actin were analyzed through immunoblotting. (B) Left panel: Raji cells were pretreated with 5-aza-CdR (10 μM) for 19 hrs; then, the cells were exposed to MG132 (10 μM) for 5 hrs. AID was immunoprecipitated, and AID ubiquitination was examined through immunoblotting. Right panel: Raji cells were treated with 5-aza-CdR (5-10 μM) for 48 hrs, and the nuclear and cytosolic extracts were harvested. The protein expression levels of AID, tubulin, and lamin A/C were analyzed through immunoblotting. (C) Upper-left panel: K562AID4 cells were treated with 5-aza-CdR (10-50 μM) and Zeb (50 μM) for 48 hrs. Lower-left panel: K562AID4 cells were treated with 5-aza-CdR (10 μM) and Zeb (10 μM) for 48 hrs, and the nuclear and cytosolic extracts were harvested. The protein expression levels of flag-AID and actin were analyzed through immunoblotting. Right panel: K562AID4 cells were treated with 10 μM DNMT inhibitors for 4 days. The cells were fixed and stained with an anti-fag antibody and DAPI and subjected to confocal microscopy analysis. (D) K562AID4 cells were treated with 5-aza-CdR (10 μM), LMB (5 ng/ml), or both for 48 hrs. The cells were fixed and stained with an anti-fag antibody (yellow) and DAPI (blue) and subjected to confocal microscopy analysis. The images are shown at 1X and 3X magnification.

AID stability has been reported to be regulated through the proteasome degradation pathway [23]. To investigate how 5-aza-CdR downregulates AID, the cells were treated with 5-aza-CdR in the presence of the proteasome inhibitor MG132. Restoration of AID expression was observed (Fig. 3A, upper panel), suggesting the involvement of proteasomal degradation in this event. To further confirm this observation, AID protein stability was examined in the presence of cycloheximide. As shown in Figure 3A, lower panel, 5-aza-CdR reduced AID protein stability, which was reversed by MG132. Because proteasome degradation is usually triggered by polyubiquitination [23], nuclear AID ubiquitination was analyzed using an immunoprecipitation assay. The smear blotting was more intense after co-treatment with 5-aza-CdR and MG132 (Fig. 3B, left panel), indicating that 5-aza-CdR enhanced AID polyubiquitination. AID degradation has been reported to occur in the nucleus [23]; therefore, nuclear AID expression was examined. AID was substantially downregulated in the nucleus by 5-aza-CdR (Fig. 3B, right panel). To confirm this finding, AID-negative CML K562 cells were transfected with flag-AID through electroporation, and stable clones were established. Nuclear flag-AID was downregulated by 5-aza-CdR but not Zeb, although total flag-AID was increased by 5-aza-CdR and Zeb (Fig. 3C, left panel). Immunofluorescence (IFA) also indicated the degradation of nuclear AID by 5-aza-CdR (Fig. 3C, right panel). Because AID is normally restricted in cytoplasm to prevent off-target deamination [24], a Crm1 inhibitor, leptomycin B (LMB), which accumulates AID in the nucleus [25], was further utilized to demonstrate the occurrence of this event in the nucleus (Fig. 3D).

### The role of AID expression in the cytotoxicity of DNMT inhibitors

**Figure 4:**
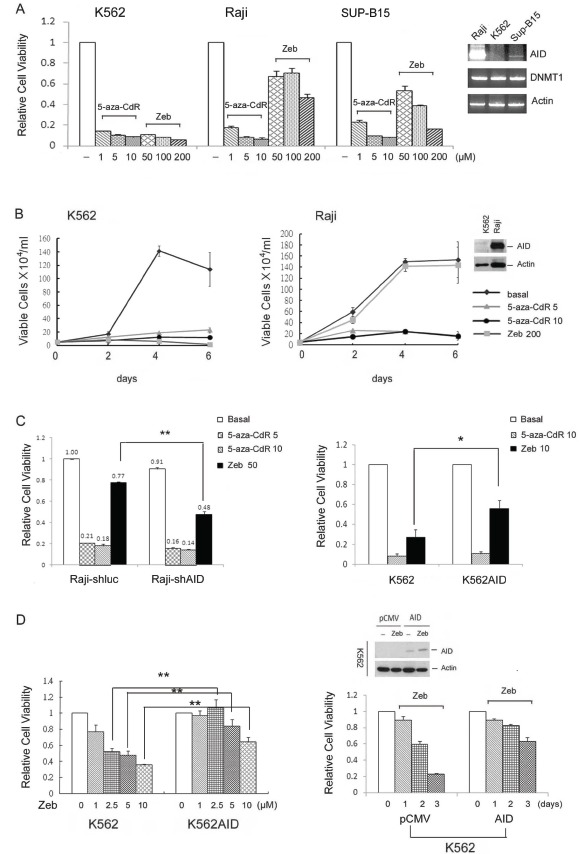
AID interferes with the cytotoxic effect of DNMT inhibitor *in vitro* (A) K562, Raji, and SUP-B15 cells were treated with 5-aza-CdR (1-10 μM) or Zeb (50-200 μM) for 4 days, and cell viability was analyzed with the Alamar blue assay. Error bars indicate the mean ± SD of three independent experiments. (B) K562 and Raji cells were treated with 5-aza-CdR (5-10 μM) and Zeb (200 μM) for 0-6 days, and cell viability was analyzed with the trypan blue assay. (C) Left panel: Raji-shluc and Raji-shAIDH1 cells were treated with 5-aza-CdR (5-10 μM) or Zeb (50 μM) for 4 days, and cell viability was analyzed with the Alamar blue assay. **, p<0.01. Right panel: K562 and pooled K562AID cells were treated with 5-aza-CdR (10 μM) or Zeb (10 μM) for 4 days. Then, cell viability was analyzed with the Alamar blue assay. *, p<0.05. (D) Left panel: K562 and pooled K562AID cells were treated with Zeb (1-10 μM) for 4 days. Then, cell viability was analyzed with the Alamar blue assay. *, p<0.01. Right panel: K562 cells were transiently transfected with pCMV or pCMV-AID plasmids and exposed to Zeb (5 μM) for 3 days. The protein expression levels of flag-AID and the cell viability were examined through immunoblotting and the Alamar blue assay, respectively.

Because 5-aza-CdR but not Zeb was found to induce AID degradation, the cytotoxicity of each agent was examined in hematopoietic cells expressing different AID levels. The cell viability of K562 (AID negative), Raji (high AID) and SUP-B15 (low AID) cells was assessed. Both 5-aza-CdR and Zeb inhibited the cell viability of K562 cells, while Raji and SupB15 cells were sensitive to 5-aza-CdR but resistant to Zeb (Fig. 4A and 4B). This differential effect indicated that AID might play an interfering role in the anticancer effect of DNMT inhibitors. To confirm this, the growth inhibitory effects of 5-aza-CdR and Zeb were examined in AID-knockdown Raji or AID-overexpressing K562 cells. The inhibition of cell viability by Zeb was enhanced in AID-knockdown Raji cells but attenuated in AID-overexpressing K562 cells (suppl. Fig. S3, Fig. 4C and 4D), while the inhibitory ability of 5-aza-CdR was not influenced by AID expression (Fig. 4C). These results suggest that 5-aza-CdR targets both AID-positive and -negative cancer cells due to its ability to deplete AID. To exclude the possibility that AID-mediated genetic mutations alter Zeb cytotoxicity, K562 cells were transiently transfected with AID. Consistently, the cytotoxicity of Zeb was attenuated by AID overexpression (Fig. 4D, right panel).

### AID interferes with the effect of DNMT1 inhibitor through stabilizing DNMT1

**Figure 5:**
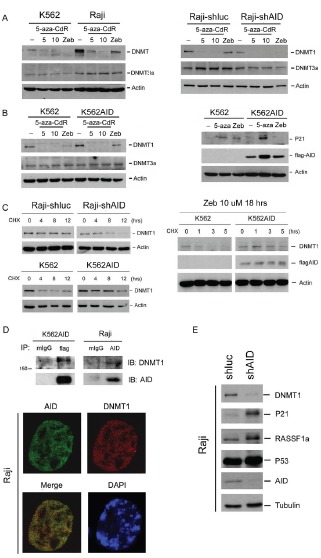
AID blocks DNMT inhibitor-induced degradation of DNMT1 and expression of TSG.s (A) Left panel: K562 and Raji cells were treated with 5-aza-CdR (5-10 μM) or Zeb (200 μM) for 24 hrs. Right panel: Raji cells transduced with the shluc or shAID plasmid were treated with 5-aza-CdR (5-10 μM) or Zeb (50 μM) for 24 hrs. (B) Left panel: K562 and pooled K562AID cells were treated with 5-aza-CdR (5-10 μM) or Zeb (10 μM) for 24 hrs. Right panel: K562 and pooled K562AID cells were treated with 5-aza-CdR (10 μM) or Zeb (10 μM) for 96 hrs. The protein expression levels of DNMTs, p21, flag-AID, and actin were examined through immunoblotting. (C) Left panel: Raji-shluc, Raji-shAID, K562 and K562AID cells were treated with cycloheximide (20 μM) for 4, 8, and 12 hrs. Right panel: K562 and K562AID cells were pretreated with Zeb (10 μM) for 18 hrs and then exposed to cycloheximide (20 μM) for 1, 3, and 5 hrs. The protein expression levels of DNMT1, flag-AID, and actin were analyzed through immunoblotting. (D) Upper panel: AID and DNMT1 were co-immunoprecipitated from the total lysate of K562AID4 cells. Flag-AID was precipitated using the anti-fag antibody. Normal mouse IgG (mIgG) was used as the IP control. The protein expression levels of DNMT1 and AID were analyzed through immunoblotting. Lower panel: Raji cells were fixed and stained with anti-AID antibody (green), anti-DNMT1 antibody (red) and DAPI (blue) and subjected to confocal microscopy analysis. (E) Proteins were harvested from Raji-shluc and Raji-shAIDH1 cells, and protein expression levels of DNMT1, p21, Rassf1a, p53, AID, and tubulin were examined through immunoblotting.

DNMT inhibitors induce growth arrest by downregulating DNMT1 to promote the expression of tumor suppressor genes [15]. Because AID expression differentially influenced the cytotoxic effect of 5-aza-CdR and Zeb, its role in DNMT1 inhibition was further examined. Both 5-aza-CdR and Zeb depleted DNMT1 in AID-deficient K562 cells, whereas the effect of Zeb was apparently attenuated in AID-positive Raji cells (Fig. 5A, left panel). Consistently, Zeb effectively downregulated DNMT1 in AID-knockdown Raji cells but not in AID-overexpressing K562 cells (Fig. 5A, right panel and 5B, left panel), indicating that the inhibitory effect of Zeb on DNMT1 might be attenuated by endogenous AID levels due to its inability to deplete AID. Similarly, the induction of the tumor suppressor gene p21 by Zeb but not 5-aza-CdR was diminished in AID-overexpressing K562 cells (Fig. 5B, right panel).

A positive correlation between the protein but not mRNA expression levels of AID and DNMT1 was observed in several stable clones of AID-knockdown Raji cells and AID-overexpressing K562 cells (suppl. Fig. S3A-S3C). AID overexpression also increased ectopic DNMT1 expression, indicating the positive regulation of these two enzymes at the protein level (suppl. Fig. S3D). Furthermore, DNMT1 stability was decreased in AID-knockdown Raji cells but increased in AID-overexpressing K562 cells (Fig. 5C, left panel), and the inhibition by Zeb was attenuated by AID overexpression (Fig. 5C, right panel). These results indicate that AID may stabilize DNMT1 to prevent its degradation by DNMT inhibitors. Flag-AID ectopically expressed in K562 cells and endogenous AID in Raji cells were co-immunoprecipitated with DNMT1 (Fig. 5D, upper pamel), and the co-localization of AID and DNMT1 in Raji cells was demonstrated (Fig. 5D, lower panel). These results suggest that the association of AID and DNMT1 might stabilize DNMT1. The silencing of AID by shAID caused DNMT1 depletion and induction of TSGs, such as p21 and Rassf1a (Fig. 5E).

### 5-aza-CdR inhibits cell growth in a mouse model

**Figure 6:**
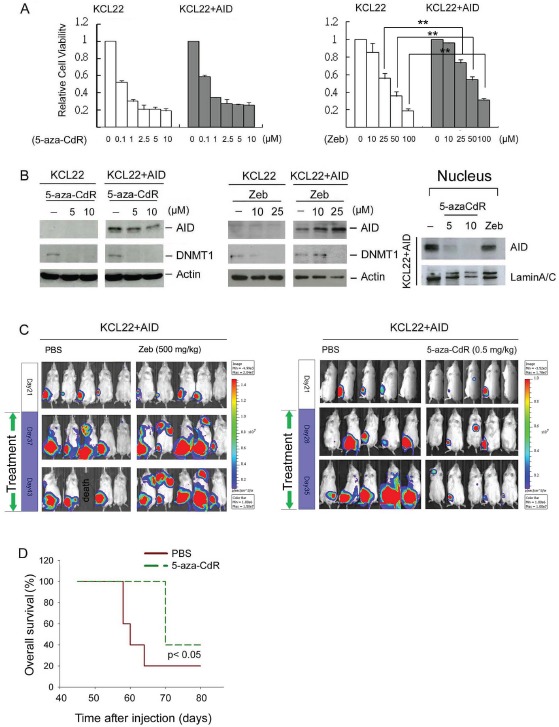
The anti-cancer effect of DNMT inhibitors on tumor-engrafted mice (A) KCL22 and KCL22+AID cells were treated with 5-aza-CdR (0.1-10 μM, left panel) and Zeb (10-100 μM, right panel) for 4 days, and cell viability was analyzed with the Alamar blue assay. The error bars represent the mean ± SD. **, p<0.01. (B) Left panel: KCL22 and KCL22+AID cells were treated with 5-aza-CdR (5-10 μM, left panel) and Zeb (10-25 μM, right panel) for 48 hrs. The protein expression levels of AID, DNMT1, and actin were analyzed through immunoblotting. Right panel: KCL22+AID cells were treated with 5-aza-CdR (5 and 10 μM) and Zeb (50 μM) for 4 days, and the nuclear extracts were harvested. The protein expression levels of AID and lamin A/C were examined through immunoblotting. (C) Left panel: Firefly luciferase-labeled KCL22+AID cells were i.t. injected into NOD/SCID recipient mice. After 21 days, tumor-bearing mice were selected and treated with PBS (n=5) or Zeb (500 mg/kg, n=5). Cell growth was examined using an in vivo imaging system (IVIS) at day 21, 37, and 43. Right panel: Firefly luciferase-labeled KCL22+AID cells were i.t. injected into NOD/SCID mice, and after 21 days, the mice were then treated with PBS (n=5) or 5-aza-CdR (0.5 mg/kg, n=5) for 21 days. Cell growth was examined using IVIS at day 21, 28, and 35. (D) Then the overall survival was depicted with Kaplan-Meier analysis.

Because AID accelerates CML progression by causing imatinib resistance [6], the effect of DNMT inhibitors on KCL22+AID cells was investigated. Consistent with the results from K562 cells, Zeb- but not 5-aza-CdR-induced cytotoxicity and DNMT1 downregulation were partially attenuated by AID overexpression, and 5-aza-CdR also downregulated nuclear AID levels (Fig. 6A and 6B). KCL22 cells harboring AID fused to a luciferase reporter were intra-tibially injected into NOD/SCID mice to examine the in vivo anticancer effect of 5-aza-CdR and Zeb. After transplantation, the cells grew in the bone marrow and spread throughout the body; However, KCL22+AID cells displayed higher growth rate (83%) than KCL22 cells (42%) (suppl. Fig. S4). The tumor-bearing mice were then selected and treated with Zeb (500 mg/kg) or 5-aza-CdR (0.5-5 mg/kg). Zeb inhibited the tumor growth of KCL22-engrafted but not KCL22+AID-engrafted mice (suppl. Fig. S5A and Fig. 6C, left panel). On the contrary, 5-aza-CdR effectively inhibited tumor growth of both KCL22 and KCL22+AID grafts (suppl. Fig. S5B and Fig. 6C, right panel), and extended the mouse lifespan (Fig. 6D). These data indicate that 5-aza-CdR but not Zeb could treat CML-LBC and AID-positive hematopoietic malignancies.

## DISCUSSION

For the past decade, targeted therapy has developed and become dominant for cancer treatment. Nevertheless, cancers are still a cureless disease because of drug resistance, which has never been resolved. Recently, substantial evidences have indicated that AID not only contributes to tumor formation and progression but also causes drug resistance by mutating tumor suppressor genes and oncoproteins [6, 10]. In addition, AID correlates with poor prognosis [26, 27]. Therefore, AID might be a potential target for preventing cancer progression and drug resistance. In this study, we found that AID interacted with and may stabilize DNMT1. This association interfered with the ability of a DNMT inhibitor to deplete DNMT1 and induce p21. 5-aza-CdR inhibited nuclear AID expression through ubiquitin-proteasome degradation, thus exerting a cytotoxic effect in AID-positive hematopoietic cancers. In contrast, Zeb effects were limited due to its inability to inhibit AID expression.

Our data have indicated that AID might interact with DNMT1 and stabilize its expression (Fig. 5 and suppl. Fig. S3). However, how AID and DNMT1 interact with each other is currently unclear. DNMT1 transfers methyl groups into the CpG islands soon after replication to maintain DNA methylation patterns in the newly synthesized single strand. Direct binding of DNMT1 to proliferating cell nuclear antigen (PCNA) in DNA replication sites has been reported[28, 29]. We found the association of AID with PCNA (suppl. Fig. S6). Therefore, PCNA might act as a bridge for AID and DNMT1 interaction. In addition, H3K9 trimethylation by G9a creates a binding platform for HP1, which recruits DNMT1 and increases DNA methylation [30]. It has been reported that AID forms a complex with KRAB domain-associated protein 1 (KAP1) and HP1 during CSR, leading to AID recruitment to switch regions [31]. Therefore, HP1 might also act as a bridge or scaffold for AID and DNMT1 interaction. It has been also shown that post-translational modifications of DNMT1 modulate its stability. SET7/9, a histone methyltransferase, mediates DNMT1 methylation at Lys142 and Lys1094 to induce its proteasomal degradation [32, 33]. In contrast, DNMT1 can be stabilized by Akt1 phosphorylation at Ser143 which inhibits Lys142 methylation [33, 34]. PKA phosphorylation of AID at Ser38 is crucial for its activity in CSR [35]. Whether PKA phosphorylation of DNMT1 at Ser143 occurs through AID/DNMT1 complex remains to be investigated.

AID-/- ALL show markedly distinct gene expression patterns, with no downregulation of TSGs, such as Rhon, p21, Blnk and TP53 in AID-/- ALL, suggesting that AID may alter gene expression patterns to increase leukemia malignancy [10]. Indeed, the ectopic expression of AID in CML cells increased cell survival in vitro and in vivo (suppl. Fig. S4 and S7). Its effect on gene instability could not fully explain this event. Our results showed that silencing AID depleted DNMT1 with the concomitant induction of hypermethylated TSGs, such as p21 and Rassf1a, suggesting that differential gene expression in AID+/+ and AID-/- cells may be attributed to DNMT1 levels in cells, in which the methylation status of tumor suppressive genes was altered. In addition, most of B cell lymphoma t(14;18) translocations occur in the CpG-rich sites where both DNA methylation and AID are required for creating the breakpoints for DNA recombination [36]. The positive association of AID and DNMT1 may be also crucial for t(14;18) translocations.

AID expression is regulated at the transcriptional and post-transcriptional levels [37]. Our results showed that AID mRNA levels were not significantly affected by 5-aza-CdR in hematopoietic cancers. Instead, 5-aza-CdR reduced the protein stability of AID by promoting its degradation in the nucleus, suggesting that 5-aza-CdR downregulated AID expression at the post-transcriptional level. Nuclear AID has been shown to be polyubiquitinated and degraded by proteasomes through an unclear mechanism [23]. 5-aza-CdR induces the degradation of DNMT1 through APC/C^Cdh1^-mediated polyubiquitination [14]. Whether APC/C^Cdh1^ E3 ligase is also involved in the AID polyubiquitination and degradation induced by 5-aza-CdR requires investigation. Recently, the E3 ligase RING finger protein 126 (RFN126) has been found for the ubiquitination of AID [38]. Whether 5-aza-CdR could upregulate RFN126 to degrade AID will also be investigated. Alternatively, AID can undergo ubiquitin-independent protein degradation through the REGγ proteasome pathway [39]. Although 5-aza-CdR-induced polyubiquitination of AID has been demonstrated, an ubiquitin-independent pathway cannot be excluded.

The structures and metabolisms of 5-aza-CdR, 5-aza-CR and Zeb are different. 5-aza-CdR is phosphorylated by deoxycytidine kinase and other kinases into triphosphate, which can be incorporated into newly synthesized DNA [40]. In contrast, 5-aza-CR and Zeb are primarily phosphorylated by uridine-cytidine kinase and other kinases into triphosphates, which are ultimately incorporated into RNA [41]. However, the diphosphate forms of 5-aza-CR and Zeb can also be reduced by ribonucleoside reductase into deoxy-diphosphates, which can be incorporated into DNA [42, 43]. Degradation of DNMT1 by 5-aza-CdR and Zeb demonstrated their incorporation into DNA [13, 44]. However, only 5-aza-CdR could trigger AID degradation. Molecular docking analyses showed that azacytidine but not Zeb-substituted ssDNA could insert into the catalytic site of AID, which might explain the differential effect of 5-aza-CdR and Zeb on AID expression.

5-aza-CR and 5-aza-CdR have been used for the treatment of MDS [45]. They have also been considered powerful candidates for acute myeloid leukemia (AML), CML and ALL [46]. Phase II studies have shown that low doses of 5-aza-CdR exhibit clinical activity against CML, including imatinib-resistant cases [47, 48] . Furthermore, the combination of 5-aza-CdR and imatinib is well tolerated and active for CML patients in the accelerated or myeloid blastic phase [49]. 5-aza-CdR is currently in a phase I clinical trial for refractory and relapsed ALL (ClinicalTrials.gov identifier: NCT00349596). Because AID contributes to tumorigenesis, imatinib resistance, clonal evolution, and immune evasion in various hematopoietic malignancies [6, 10, 50], our results provide a novel molecular basis for a new indication of 5-aza-CdR in treating AID-positive hematopoietic cancers.

**Figure 7:**
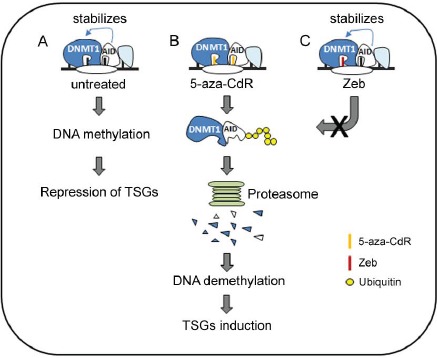
A hypothetical model for the role of AID in the anticancer effect of DNMT inhibitors (A) AID may co-localize with DNMT1 on DNA and enhance its stability in B-lymphoid malignancies. 5-aza-CdR and Zeb incorporate into DNA. (B) 5-aza-CdR targets the active site of AID and DNMT1 and then destabilizes AID and DNMT1 through the proteasome degradation pathway. Eventually, DNA is demethylated, and TSGs are induced. (C) Because Zeb is unable to bind to the active site of AID, DNMT1 is stabilized and avoids degradation by Zeb. Thus, the anticancer effect of Zeb is inhibited

Taken together, we propose a model for our hypothesis (Fig. 7). The incorporation of DNMT inhibitors into DNA triggers DNMT1 degradation through the proteasomal pathway, resulting in DNA demethylation, TSG induction, and growth arrest. In AID-overexpressing hematopoietic cancer cells, however, AID interacts with and stabilizes DNMT1, which blocks the anticancer effect of Zeb due to its inability to downregulate AID and hinders DNMT1 degradation. In contrast, DNA-incorporated 5-aza-CdR can trigger AID degradation through an ubiquitin-proteasome pathway. Thus, 5-aza-CdR can effectively degrade DNMT1 to exert its anti-cancer effect against both AID-positive and AID-negative cells. Our results provide a novel role for the clinical utility of 5-aza-CdR to treat AID-expressing cancers and indicate the crucial concern for the selection of DNMT inhibitors. AID downregulation by DNMT inhibitors, such as 5-aza-CdR, may be beneficial for the treatment of AID-expressing cancers.

## MATERIALS AND METHODS

### Cell culture and construction of stable clones

AID-overexpressing CML KCL22+AID cells (received from Dr. Markus Müschen, University of California San Francisco) and ALL SUP-B15 cells (obtained from Dr. Hu, Med. Biotech, National Taiwan University) were cultured in IMDM. The Burkett's lymphoma Raji cells (received from Dr. Doong, Department of Microbiology, National Taiwan University) and the CML K562 cells were maintained in RPMI 1640 medium. All media were supplemented with 10% fetal bovine serum (Gibco), 1% L-glutamine (Gibco), and 1% antibiotic:antimycotic solution (Gemini Bio Products), and the cells were incubated at 37°C in a humidified incubator containing 5% CO_2_. For the generation of stable clones, the pLKO.1-shAID plasmid (purchased from the National RNAiCore Facility, Academia Sinica, Taipei, Taiwan) was transduced into Raji cells through lentivirus infection, and Raji-shAID cells were selected using 100 ng/ml puromycin. The pCMV-3XFLAG-AID plasmid (received from Dr. Riccardo Dalla-Favera, Department of Microbiology, Columbia University) was transfected into K562 cells through electroporation (Nero Transfection System, Invitrogen), and K562AID cells were selected with 1 mg/ml G418 (Gibco). 5-azaCR (Sigma Aldrich), 5-aza-CdR (Biovision) and zebularine (Sigma Aldrich) for in vitro experiments were prepared in DMSO at appropriate doses. Zebularine (NSC 309132) for animal studies was provided by the Drug Synthesis and Chemistry Branch, Developmental Therapeutics Program, Division of Cancer Treatment and Diagnosis, National Cancer Institute.

### Homology modeling

The sequence alignment between AID and APOBEC-2 was performed with the ClustalW module using the BLOSUM multiple alignment scoring matrix in Discovery Studio 2.55. The sequence identity and similarity between AID and APOBEC-2 were 31.8% and 51.0%, respectively. The homology model structure was generated and optimized using MODELLER in Discovery Studio 2.55. The APOBEC-2 structure was obtained from the Protein Data Bank (PDB ID: 2NYT). The structure with the lowest energy score was selected as the final model.

### Molecular docking

Docking simulations of single-stranded DNA were performed using PLANTS 1.2 software. The structure of single-stranded DNA (5'-A-T-azaC-G-3') was built with the “Build and Edit Nucleic Acid” tool in Discovery Studio 2.55. The binding site was set as a sphere with a 40 Å radius centered from the zinc atom to ensure that the entire protein structure was included. All parameters were set to the default settings. Docking simulations of the small molecules Zeb and 5-aza-CdR were performed using the CDOCKER program in Discovery Studio 2.55 with the default parameters. Ligand structures were constructed using the ChemDraw Ultra 12 program and further processed using the Prepare Ligands protocol in Discovery Studio 2.55 to convert to a 3D structure and assign the ionization state. All parameters were set to the default settings.

### Immunofuorescence (IFA) and immunoblotting

For IFA, the slides with cells were immersed in 2% paraformaldehyde for 20 min. After fixation, TBST with 1% goat serum was used to block the slides. The cells were first incubated with the primary antibody anti-fag (Sigma Aldrich) or co-incubated with anti-AID (Cell Signaling) and anti-DNMT1 at 4°C overnight; then, the secondary antibodies Alexa (488)-labeled anti-mouse and Alexa (647)-labeled anti-goat (Invitrogen) were added and incubated at 25°C for 30 min. The signal was observed using a confocal microscope (Leica SP5). For immunoblotting, the cells were lysed in sample buffer (50 mM Tris, 1 mM EGTA, 50 mM NaF, 150 mM NaCl, 1 mM Na_3_VO_4_, 1 mM β-glycerophosphate, 8.5 mM sodium pyrophosphate, and 0.5% Triton X-100). lysates were resolved on a 7.5% or 13% sodium dodecyl sulfate-polyacrylamide gel followed by electrotransference to a nitrocellulose membrane (Hybon-C). Then, the membranes were incubated in TBST containing 5% milk for 1 hr. Ultimately, the membranes were incubated with the following primary antibodies: anti-DNMT1, anti-DNMT3a, anti-ubiquitin, anti-p21, anti-lamin A/C (all from Santa Cruz Biotechnology), anti-actin (Millipore), anti-tubulin (Sigma-Aldrich) or anti-AID (Cell Signaling).

### Immunoprecipitation

Total cell lysates or nuclear extracts were diluted to 1 μg/μl, and 200-500 μl lysate was incubated with 2-5 μl anti-AID (Cell Signaling) or anti-fag antibody (Sigma Aldrich) overnight at 4°C. The mixtures containing the lysates and antibody were added to 50% protein-A beads at a 1:20 ratio (volume) for 1 hr at 4°C; then, the beads were precipitated at 2000 rpm for 5 min. Then the beads were washed with PBS three times to clear nonspecific proteins. A total of 20 μl PBS and 4 μl 6X sample dye were mixed with beads to prepare samples for immunoblotting.

### *In vivo* imaging system (IVIS)

KCL22+AID-luc cells (5x10^5^) were intra-tibially (i.t.) injected into NOD/SCID mice. After the xenograft, the mice were imaged at different time points using IVIS (Xenogen, Caliper). The successfully transplanted mice were selected to examine the anticancer effect of the DNMT inhibitors. The treatments were administered weekly on 5 consecutive days as 300 μl i.p. injections of PBS, 5-aza-CdR (0.5 mg/kg) or Zeb (500 mg/kg). D-luciferin (Promega) dissolved in PBS was injected into mice at a dose of 2.5 mg/mice, and the light emission was measured 1 min later. For anesthesia, 2.5% isoflurane was administered to the mice via a nose cone. All animal procedures were performed under protocols approved by the Institutional Animal Care and Use Committee of the College of Medicine, National Taiwan University.

### RT-PCR

Total RNA was extracted from cells using TRI_ZOL_ Reagent (Invitrogen). RNA (2 μg) was reverse transcribed into 20 μl cDNA by Moloney Murine Leukemia Virus Reverse Transcriptase (M-MLV RT) (Promega) at 420c for 1 hr, and then the PCR was performed using Taq polymerase (Geneaid). PCR products were resolved in 1% agarose gel and visualized by Gel Doc 200 (Bio Rad). The oligonucleotide primers for PCR amplifcation were as followed: AID, 5'-AGGCAAGAAGACACTCTGGACACC-3' (forward), 5'-GTGACATTCCTGGAAGTTGC-3' (reverse), β-actin, 5'-TGACGGGGGTCACCCACTGTGCCCATCTA-3' (forward), 5'-CTAGAAGCATTTGCGGGGGACGATGGAGGG-3' (reverse). For quantitative PCR, the cDNA mixed with SYBR Green Master (Roche) was amplifed and detected by AVI 7900 (AVI). The oligonucleotide primers for quantitative PCR were as followed: AID, 5'-GGACTTTGGTTATCTTCGCAA-3' (forward), 5'-GTCGGGCACAGTCGTAGC-3' (reverse), β-actin, 5'-CCAACCGCGAGAAGATGA-3' (forward), 5'-TCCATCACGATGCCAGTG-3' (reverse).

### Cell viability assay

Raji (5 × 10^4^ cells/ml), K562 (2 × 10^4^ cells/ml) and SUP-B15 (5 × 10^5^ cells/ml) were seeding in 12 well plate, and then treated with DNMT inhibitors at indicated doses for 96 hrs. Cells (100 μl) were transferred to 96 well and 10 μl alarma blue (Invitrogen) was added to each well. After 2 hrs, cell viability was analyzed by detecting fluorescence. The wavelength at 525-535 nm was used to excite fluorescence. Reference wavelength 535-590 nm was measured on a multiwell plate reader.

### Statistical analysis

Data were analyzed using Student's *t* test. P values < 0.05 were considered significant.

## Supplementary Figures


