# Supplementary material for: AID downregulation is a novel function of the DNMT inhibitor 5-aza-deoxycytidine
Source: Oncotarget. 2013 Nov 25;5(1):211–23. doi: 10.18632/oncotarget.1319 (PMC3960202; doi:10.18632/oncotarget.1319)
Supplement: Supplementary file 1 [file oncotarget-05-0211-s001.pdf]

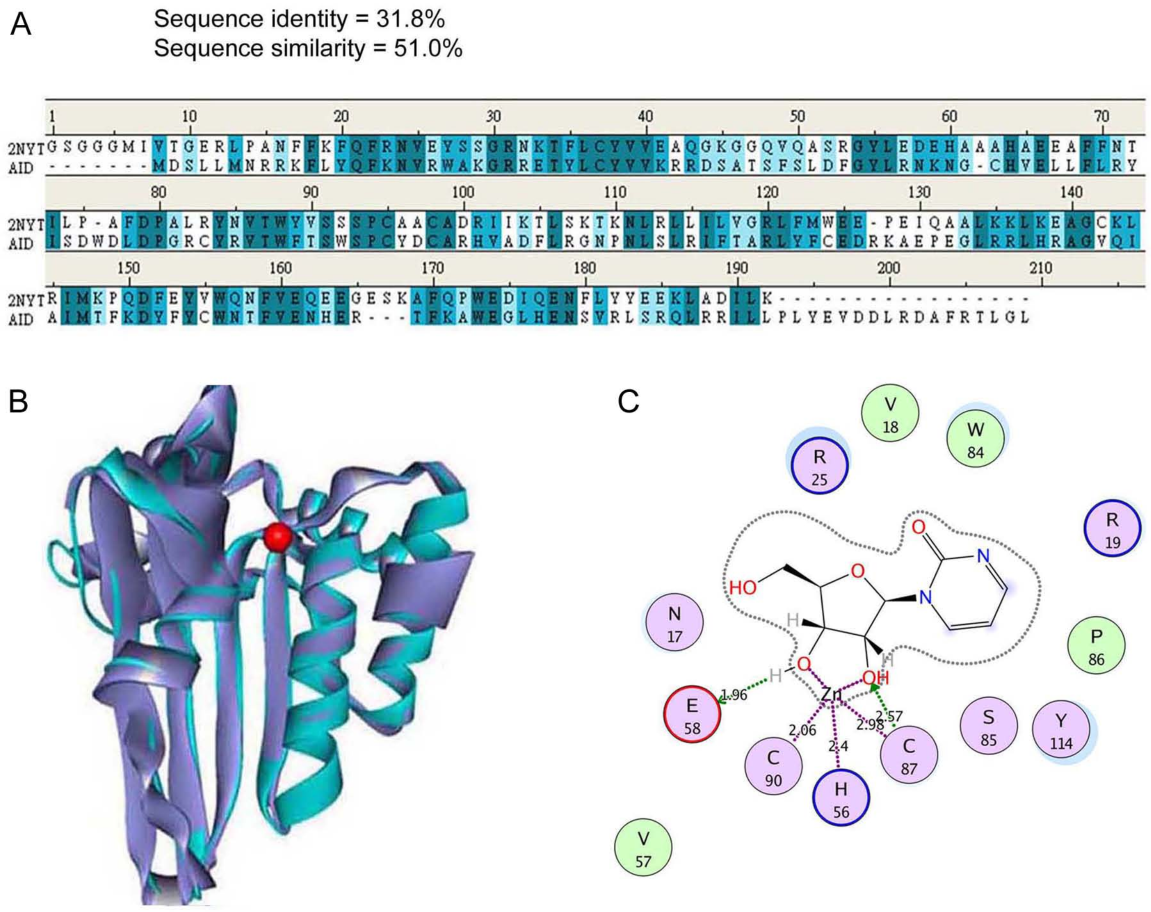

Suppl. Fig. S1. Homology modeling of AID.

(A) Sequence alignment between apobec2 (2NYT) and AID. **(B)** AID structure (green) was constructed by alignment of apopbec2 (purple). The red bead inside the pocket site represents a zinc ion. **(C)** Protein ligand interaction among the active site of AID.

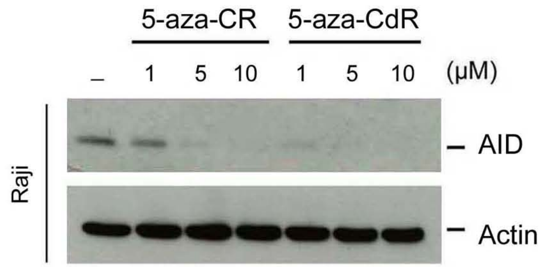

Suppl. Fig. S2. Azacytidines inhibit AID expression.

(A) Immunoblotting of AID harvested from Raji cells treated with indicated DNMT inhibitors for 96 hrs. Protein expression levels of AID and Actin were examined by immunoblotting.

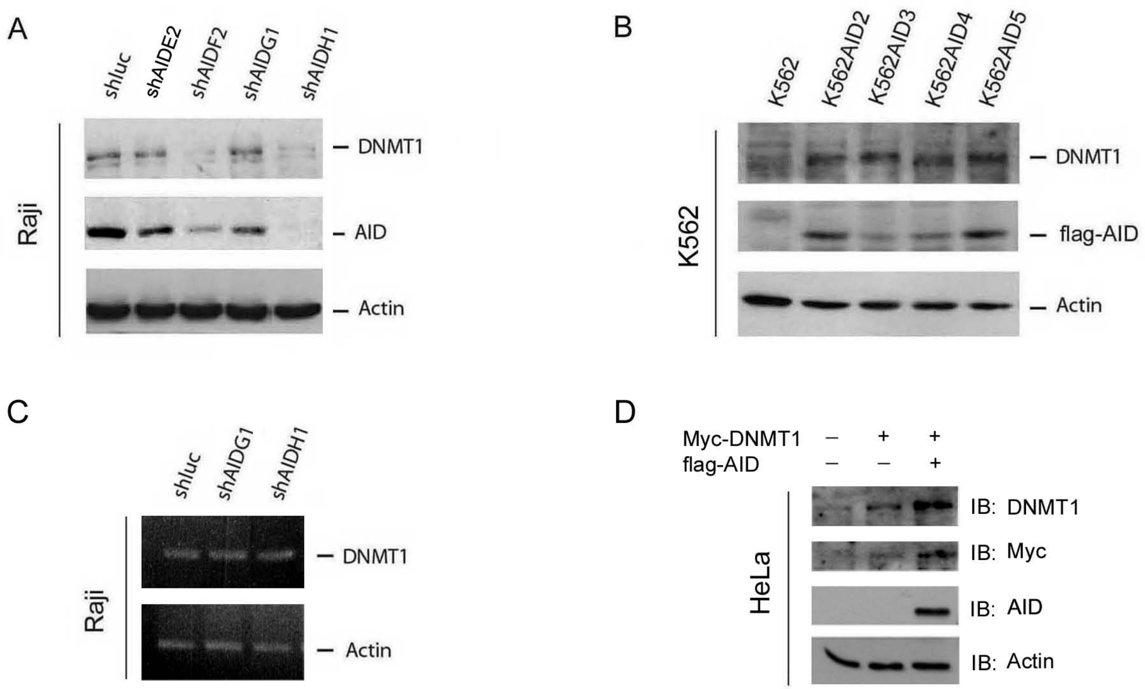

Suppl. Fig. S3. Correlation of AID and DNMT1 protein expression.

Immunoblotting of proteins harvested from Raji-shluc, Raji-shAIDs **(A)**, K562 and K562AIDs **(B)**. Protein expression of DNMT1, flag-AID and Actin was examined. **(C)** RT-PCR of mRNA harvested from Raji-shluc, Raji-shAIDs. The mRNA levels of DNMT1 and Actin were analyzed. **(D)** $1x10^6$ Hela cells were co-transfected with myc-DNMT1 (provided by Dr. Li, Graduate Institute of Biomedical Science, Chang Gung University) and pCMV-3XFLAG-AID. Protein expression levles of total DNMT1, myc-DNMT1 and flag-AID and Actin were examined.

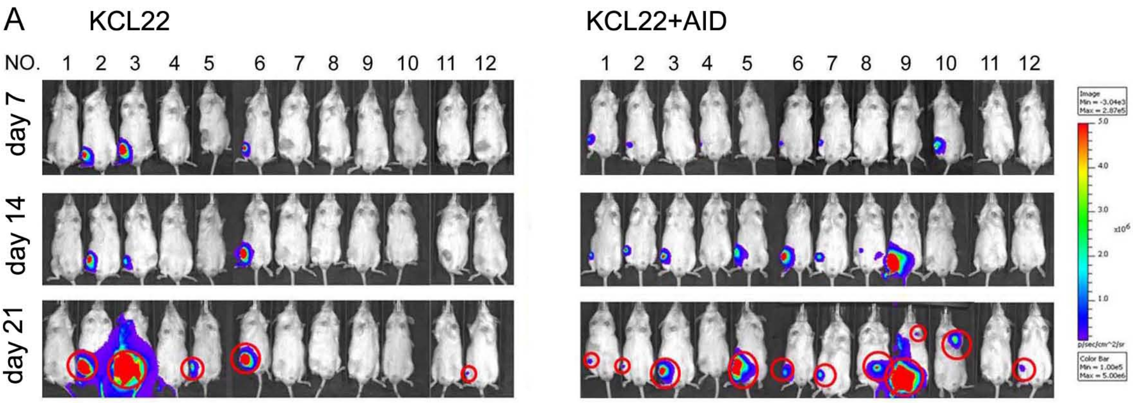

Growth rate :
KCL22 : 5/12 (42%)
KCL22+AID : 10/12 (83%)

Suppl. Fig. S4. Growth rate of CML cells in the engraft model.

$5x10^5$ Firefly lucifierase-labeled KCL22 and KCL22+AID cells were i.t. injected into 24 NOD/SCID mice, and cell growth was examined by an in vivo image system (IVIS).

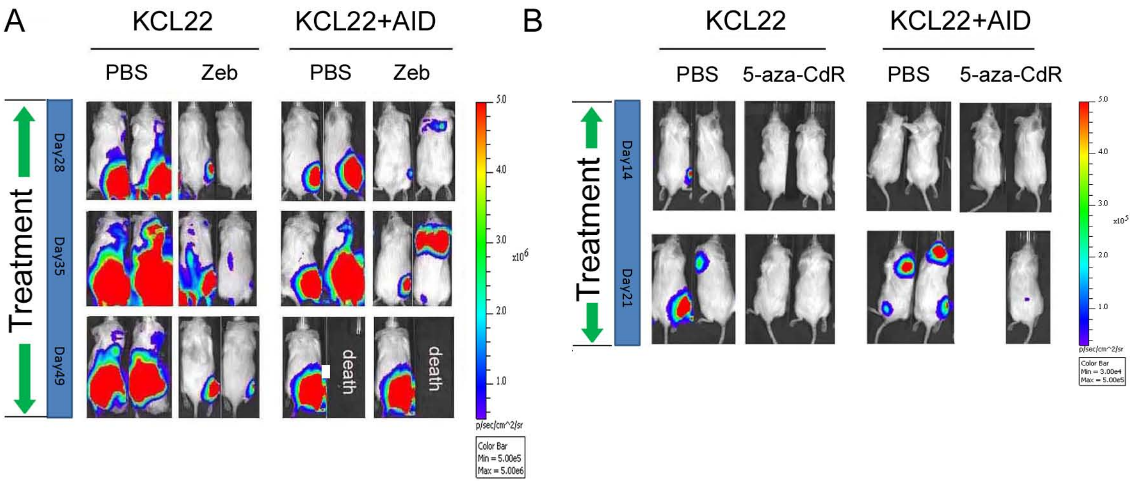

Suppl. Fig. S5. The anti-cancer effect of DNMT inhibitors in KCL22- and KCL22+AID-engrafted mice.

Firefly lucifierase-labeled KCL22 and KCL22+AID CML cells were i.t. injected into NOD/SCID recipient mice. (A) After 21 days, tumor-bearing mice were selected and treated with PBS (n=2) or Zeb (500 mg/kg, n=2). **(B)** After 7 days, tumor-bearing mice were selected and treated with PBS (n=2) or 5-aza-CdR (5 mg/kg, n=2). Leukemia Cell growth was monitored using an in vivo imaging system (IVIS).

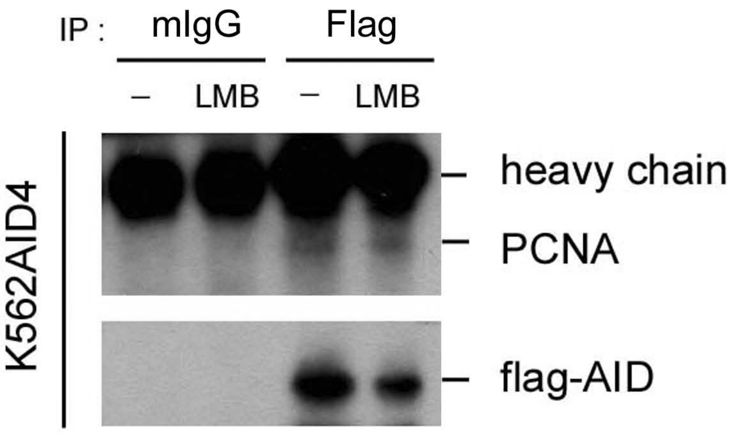

Suppl. Fig. S6. Co-IP of AID and PCNA.

AID and PCNA were co-immunoprecipitated from the total lysate of K562AID4 cells treated with LMB (5 ng/ml) for 24 hrs. Flag-AID was precipitated using the anti-flag antibody. Normal mouse IgG (mIgG) was used as the IP control.

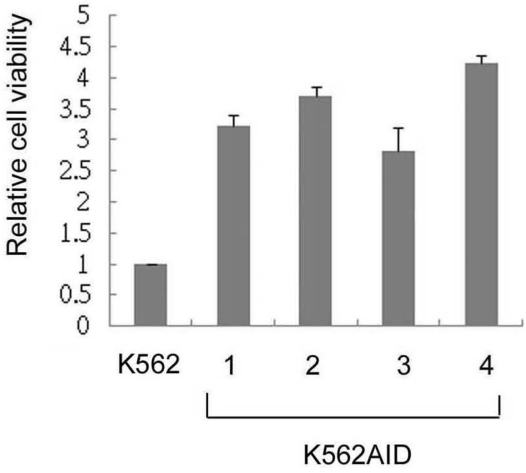

Suppl. Fig. S7. Cell viability of K562 and K562AIDs.

K562 and K562AIDs were cultured in RPMI containing 1% serum for 96 hrs. Cell viability was analyzed by alarma blue assay. Numbers indicate the different single AID expressing clones. The error bars represent mean ± SD (n = 2).
